# Supplementary material for: Interleukin (IL)-22 from IL-20 Subfamily of Cytokines Induces Colonic Epithelial Cell Proliferation Predominantly through ERK1/2 Pathway
Source: Int J Mol Sci. 2019 Jul 15;20(14):3468. doi: 10.3390/ijms20143468 (PMC6678670; doi:10.3390/ijms20143468)
Supplement: Supplementary file 1 [file ijms-20-03468-s001.pdf]

International Journal of Molecular Sciences

MDPI

Supplementary Materials

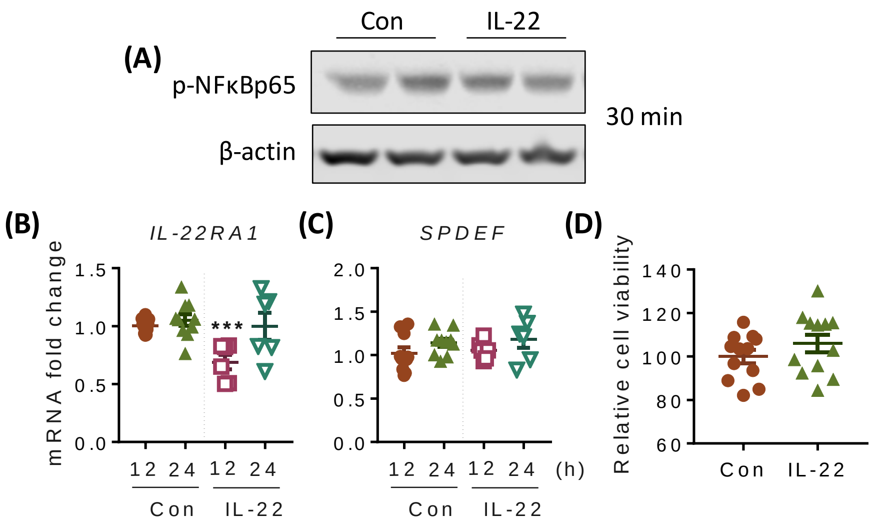

Supplementary Figure S1. Cells were treated with IL-22 (50 ng/ml) for 30 min and checked for (A) phosphorylated NFκBp65 through western blotting. Following 12 and 24 h of treatment, cells were harvested to collect RNA. Then qRT PCR were performed to check the expressions of (A) GRP78 and (B) IL-22 receptor subunit IL-22RA1 and (C) goblet cells-associated factors SPDEF. (D) at 24 h of treatment, MTT assay was performed to check any possible cytotoxicity induced by IL-22. Data are presented as mean ± SEM with individual dot points (n=6-12 from 2-3 independent experiments). Nonparametric Man-Whitney t-test was performed to check statistical significance.

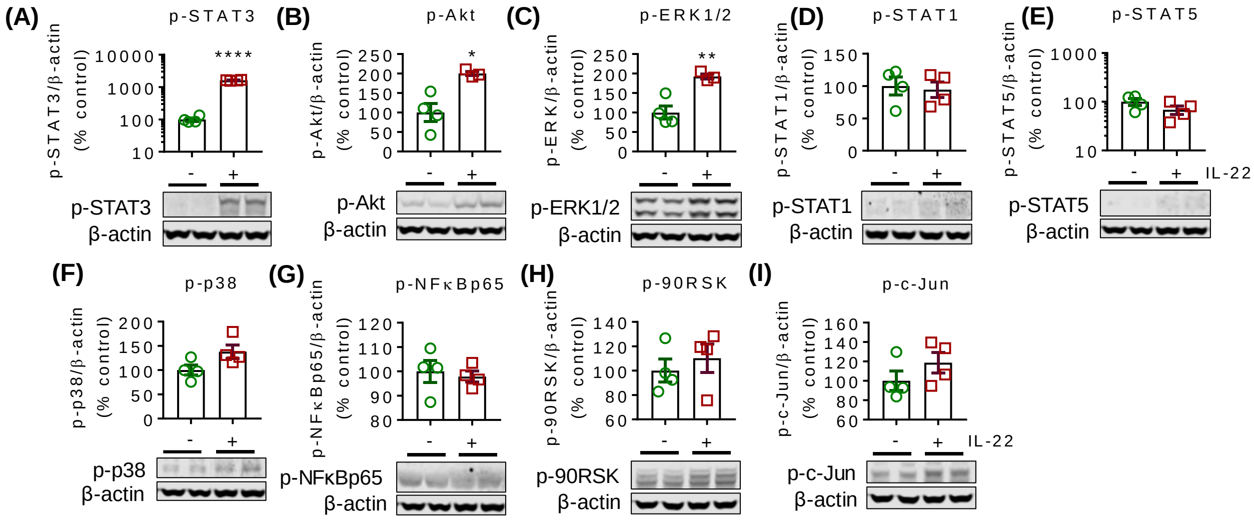

Supplementary Figure S2. mIECs were treated with IL-22 (100 ng/mL) for 1 h and checked for (A) phosphorylated STAT3, (B) Akt, (C) ERK1/2, (D) STAT1, (E) STAT5, (F) p38, (G) NFκBp65, (H) 90RSK, and (I) c-Jun through western blotting. Data are presented as mean ± SEM with individual culture from different mouse (n=4). *p<0.05; **p<0.01, ****p<0.0001 compared with control (Nonparametric Man-Whitney t-test).

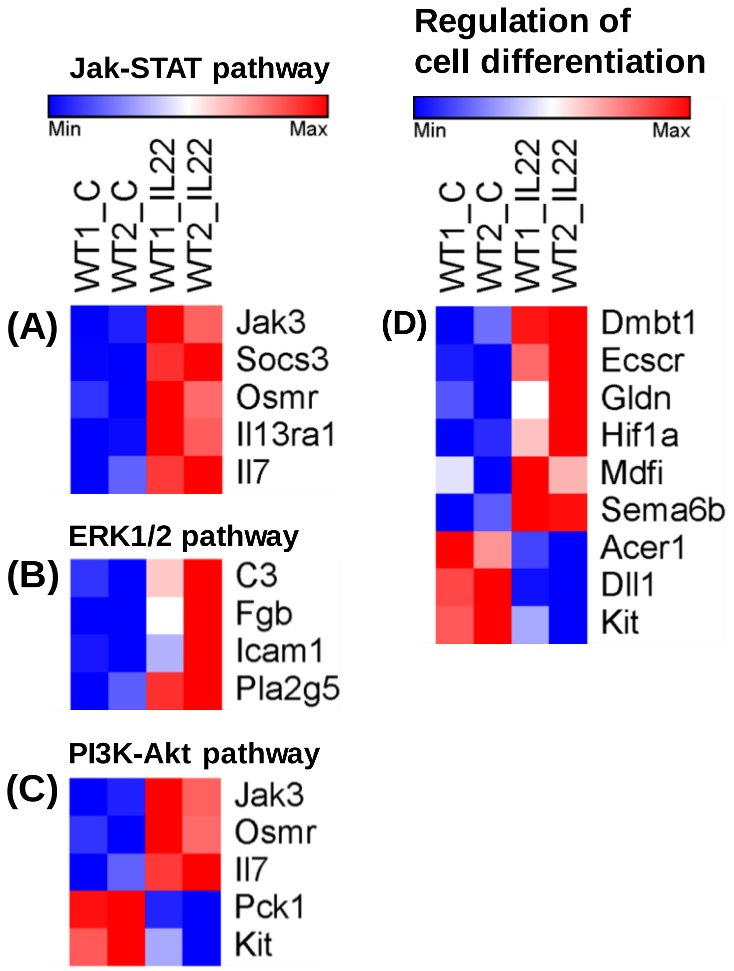

Supplementary Figure S3. mIECs were treated with 100 ng/mL of mIL-22 for 4 h. RNA-Seq was conducted and KEGG pathway analyses showed activation of (A) Jak-STAT, (B) ERK1/2, and (C) Akt pathways. (B) gene ontology analyses showed differential expression of a cluster of genes associated with regulation of cell differentiation.

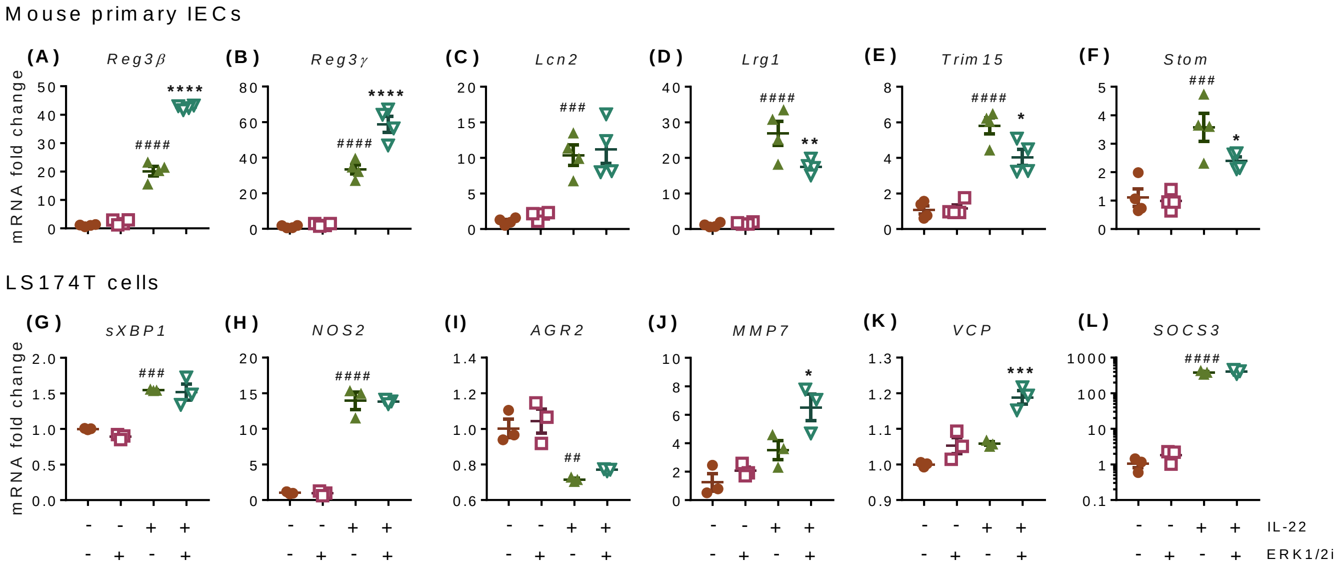

Supplementary Figure S4. Cells were pre-treated with 10 μM of ERK1/2 inhibitor for 1 h and then induced with IL-22 (100 ng/ml for 4 h in mIECs and 50 ng/ml for 12 h in LS174T cells). The harvested mRNA were then analysed through qRT PCR to check the expressions of (A) Reg3β, (B) Reg3γ, (C)

Lcn2, (D) Lrg1, (E) Trim15, and (F) Stom in mIECs and (G) ER stress marker sXBP1, (H) oxidative stress marker NOS2, (I) protein disulphide isomerase AGR2, (J) matrix metalloprotease MMP7, (K) valosin containing protein (VCP), and (L) suppressor of cytokine signaling 3 (SOCS3) in LS174T cells. Data are presented as mean ± SEM with individual dot points (n=3 from 1 experiment). ###p<0.001; ####p<0.0001 compared with control and *p<0.05; ***p<0.001compared with IL-22 (one-way ANOVA followed by Dunnett's post hoc test).

Supplementary Table S1. List of primers used in this study.

Primer	Sequence
h*β-ACTIN*	Forward: CCTGTACGCCAACACAGTGC<br>Reverse: ATACTCCTGCTTGCTGATCC
h*IL-22RA1*	Forward: TCGATTCAGATGATTGTTCA<br>Reverse: CTGATCTGGGAGTCACTGGAC
h*GRP78*	Forward: GCCTGTATTTCTAGACCTGCC<br>Reverse: TTCATCTTGCCAGCCAGTTG
hs*XBP1*	Forward: CAAAAGGATATCAGACTCAGAATCTGAA<br>Reverse: GAGTCCGCAGCAGGTGC
h*NOS2*	Forward: CACTCAGCTGTGCATCGAC<br>Reverse: CAGTTCCCGAAACCACTCGT
h*MUC2*	Forward: CAGCACCGATTGCTGAGTTG<br>Reverse: GCTGGTCATCTCAATGGCAG
h*REG3α*	Forward: AGCTACTCATACGTCTGGATTGG<br>Reverse: CACCTCAGAAATGCTGTGCTT
h*REG3γ*	Forward: GGTGAGGAGCATTAGTAACAGC<br>Reverse: CCAGGGTTTAAGATGGTGGAGG
h*AGR2*	Forward: GAGCTGTATCTGCAGGTTCGT<br>Reverse: ATTGGCAGAGCAGTTTGTCC
h*HES1*	Forward: AGTGAAGCACCTCCGGAA<br>Reverse: TCACCTCGTTCATGCACTC
h*ATOH1*	Forward: AACGCCTTGTCCGAGCTGCTA<br>Reverse: TTTTGCAGGAGGCTGGAG
h*SPDEF*	Forward: GCACTGCAGCAGACA<br>Reverse: GGGGATACGCTGCTC
h*DDR2*	Forward: GCTATATGCCGCTATCCTCTGG<br>Reverse: ACTCTGACCACTGACTGGAAG
h*LRG1*	Forward: GGACACCCTGGTATTGAAAGAAA<br>Reverse: TAGCCGTTCTAATTGCAGCGG
h*TRIM15*	Forward: TCCCTGAAGGTGGTCCATGAG<br>Reverse: CAGGATCTTGCCCGAGGATT
h*LCN2*	Forward: CCACCTCAGACCTGATCCCA<br>Reverse: CCCCTGGAATTGGTTGTCCTG
h*STOM*	Forward: CACACACGGGACTCCGAAG<br>Reverse: ATGAGAACGCCACCAAAATCC
h*MMP7*	Forward: TCGGAGGAGATGCTCACTTCGA<br>Reverse: GGATCAGAGGAATGTCCCATACC
h*VCP*	Forward: GGAGYTGGTYCAGTATCCTGTGG<br>Reverse: CAGCTTGSCGKGCCTTGT
h*SOCS3*	Forward: CCTGCGCCTCAAGACCTTC<br>Reverse: GTCACTGCGCTCCAGTAGAA
m*β-actin*	Forward: GAAATCGTGCGTGACATCAAA<br>Reverse: CACAGGATTCCATACCCAAGA

m*Ddr2*	Forward: ATCACAGCCTCAAGTCAGTGG<br>Reverse: TTCAGGTCATCGGGTTGCAC
m*Lrg1*	Forward: TTGGCAGCATCAAGGAAGC<br>Reverse: CAGATGGACAGTGTCGGCA
m*Timp1*	Forward: TACACCCCAGTCATGGAAAGC<br>Reverse: CGGCCCGTGATGAGAAACT
m*Trim15*	Forward: TGAGCGAGACCTACTGTGAAG<br>Reverse: AACCGACTCCTGAGACGATCC
m*Reg3β*	Forward: ACTCCCTGAAGAATATACCCTCC<br>Reverse: CGCTATTGAGCACAGATACGAG
m*Osmr*	Forward: TCAAGCCACGAAGGGTCCTAA<br>Reverse: GTCTTAAAGTCTCGGGTTTCACA
m*Lcn2*	Forward: TGGCCCTGAGTGTCATGTG<br>Reverse: CTCTTGTAGCTCATAGATGGTGC
m*Stom*	Forward: ATCATCTTTAGACTGGGTCGCA<br>Reverse: TGAACACGGTAGTAGACCACA
